# The Enigma of Giant Phyllodes Tumour

**DOI:** 10.7759/cureus.22946

**Published:** 2022-03-08

**Authors:** Seng Yeong Gan, Maya Mazuwin Yahya, Wan Zainira Wan Zain, Nik Fatin Amirah Nik Min, Wan Faiziah Wan Abdul Rahman

**Affiliations:** 1 Department of Surgery, School of Medical Sciences, Universiti Sains Malaysia, Kota Bharu, MYS; 2 Breast Cancer Awareness & Research Unit, Hospital Universiti Sains Malaysia, Kota Bharu, MYS; 3 Department of Pathology, School of Medical Sciences, Universiti Sains Malaysia, Kota Bharu, MYS

**Keywords:** giant phyllodes tumour, breast cancer, breast neoplasm, phyllodes tumour, breast tumour

## Abstract

Phyllodes tumours are an uncommon type of biphasic fibroepithelial neoplasm of the breast. We present a case of a 28-year-old, para one lady with no risk of breast cancer presented with painless left breast swelling for three months. Over one month, the swelling suddenly increased in size and became painful with skin changes associated with pus discharge. On physical examination, a huge swelling measuring about 25cm x 30cm occupies the central and lateral aspect of the left breast with surrounding erythema*. *We proceeded for a tru-cut biopsy, and the histopathological examination (HPE) showed a stromal proliferation with myxoid changes consistent with phyllodes tumour of benign type. The patient underwent a left simple mastectomy, and the histopathological examination (HPE) confirmed the diagnosis of borderline phyllodes tumour with clear margins without lymph nodes involvement. The patient was subsequently referred to the oncology team and was subjected to 40 Gy in 15 fractions of radiotherapy. Given the rarity of the disease and based on current studies, simple mastectomy with negative margins is recommended for giant benign phyllodes tumours

## Introduction

Phyllodes tumours are what we call an enigma in breast surgery. This tumour is an uncommon biphasic fibroepithelial neoplasm of the breast that can be classified into benign, borderline, and malignant. Phyllodes tumour consists of 0.3% to 1% of all breast tumours [1]. Benign phyllodes tumours constitute 60% to 75%, borderline 15% to 26%, whereas malignant account for 8% to 20% of cases [2]. Although the majority is benign, its variable categories cause challenges in management [3]. ﻿ The tumour size can be various and frequently large, with a median size from 4 cm to 5 cm. Giant phyllodes tumour is defined when the size is more than 10 cm [1]. ﻿Multiple terms are used to describe these tumours, such as cystosarcoma phyllodes, cellular fibroadenoma, and juvenile fibroadenoma [4]. Originally, the term cystosarcoma phyllodes were first introduced by ﻿Johannes Müller in 1838 [1,4]. It is originated from the Greek word sarcoma, which means flesh appearance, and phyllon, which means leaflike [1,4].

## Case presentation

**Figure 1:**
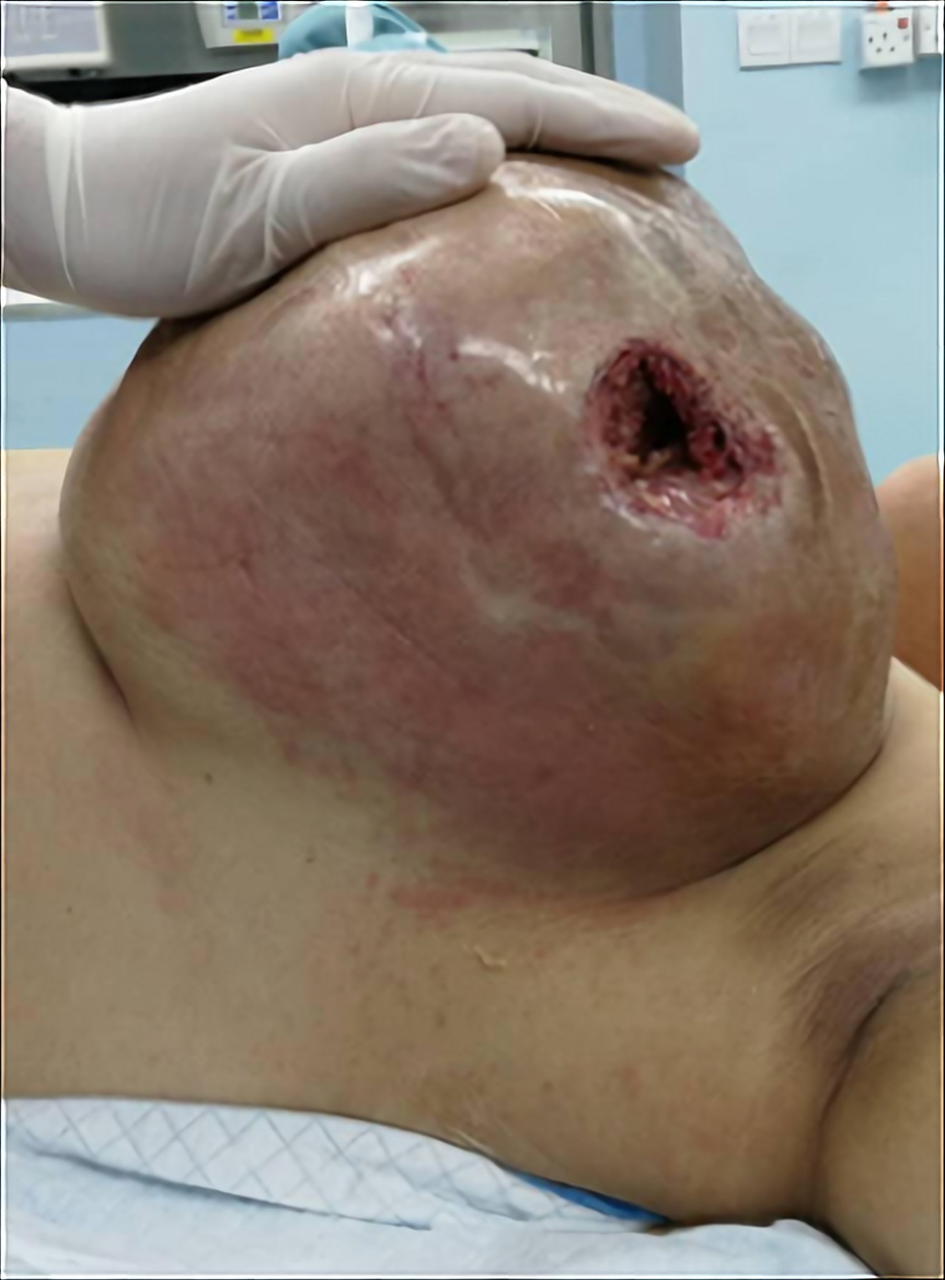
Large punch-out ulcer at the lateral aspect of the left breast tumour

A 28-year-old, para one lady with no risk of breast cancer presented with painless left breast swelling for three months. Over one month, the swelling suddenly increased in size and became painful with the changes over the skin and associated with pus discharge. On physical examination, there was a huge swelling measuring about 25cm x 30cm occupied the central and lateral aspect of the left breast with surrounding erythema (Figure 1). There were no palpable axillary lymph nodes. Aspiration of the pus was done and sent for culture and sensitivity. The result returned as *Staphylococcus aureus*, and the cytology examination reported no malignant cells. 

**Figure 2:**
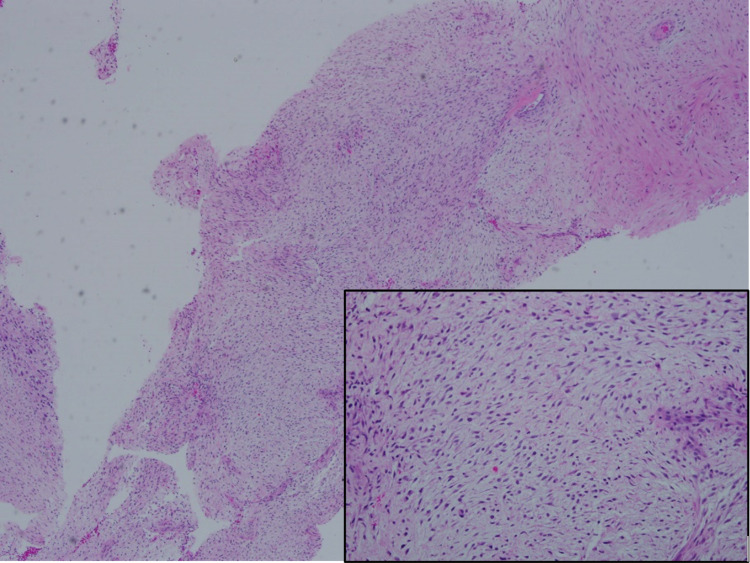
Tru-cut biopsy from the giant breast lesion shows stromal proliferation without an obvious pattern of arrangement. (Inset) Spindle cell proliferation with minimal atypia and myxoid changes (H&E, X40)

**Figure 3:**
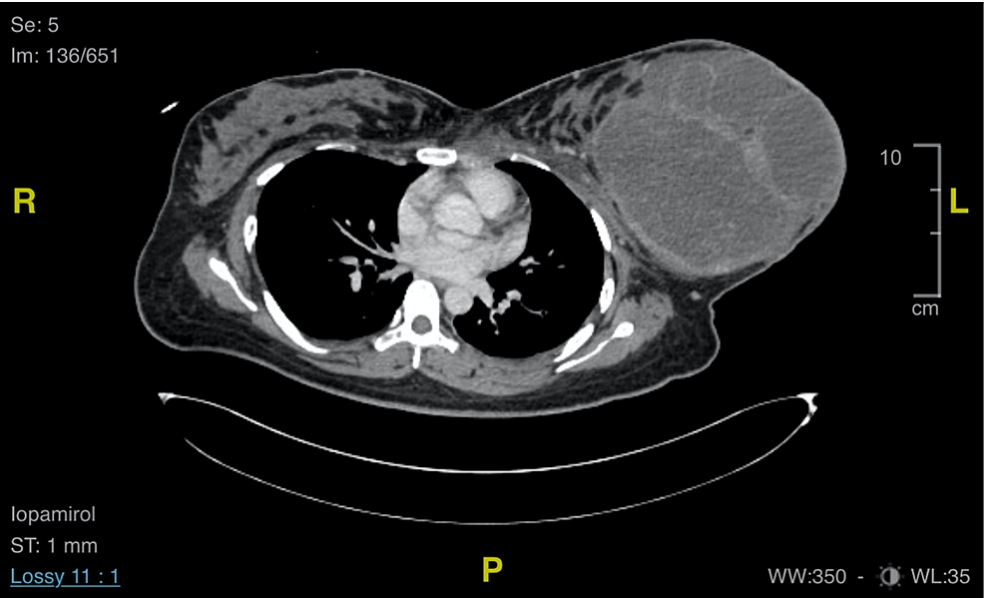
CT TAP shows a large heterogeneous left breast mass which is mainly cystic in nature with the presence of thick enhancing septations, surrounded by a thick, irregular enhancing wall with surrounding fat streakiness and nodal metastasis. CT TAP: Computed Tomography (CT) Thorax, Abdomen and Pelvis

The case then proceeded for a tru-cut biopsy. The histopathological examination (HPE) showed a stromal proliferation with myxoid changes consistent with phyllodes tumour of benign type (Figure 2). However, in view of focal atypia and mitotic figures about 3/10 hpf, the malignant phyllodes cannot be ruled out. The patient was subjected to Computed Tomography (CT) Thorax, Abdomen and Pelvis, and the report came back as a large left breast mass, most likely a phyllodes tumour with nodal metastasis (Figure 3).

**Figure 4:**
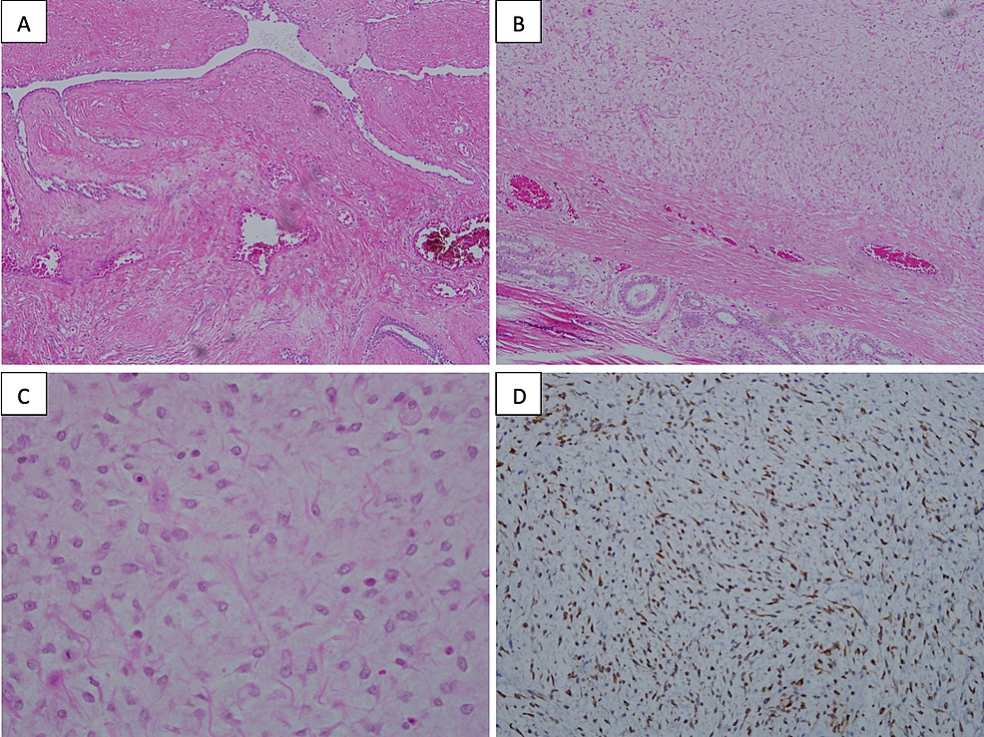
Histopathological examination from the left mastectomy A) Scattered entrapped tubular glands with exaggerated intracanalicular pattern of glands surrounded by hyaline stroma (H&E x 20). B) Spindle cell proliferation in the myxoid background with fibrous collagen bundles in between and entrapped glands (H&E x20). C) High power view of spindle cells shows fairly monomorphic with low mitotic count (H&E x 40). D) The stromal cells are diffusely positive for BCL-2 immunohistochemistry (x20)

The patient underwent a left simple mastectomy for resection of the tumour that occupied the whole left breast together with surrounding ulceration and pus discharge over the lower outer quadrant. There were no pectoralis fascia or muscle infiltration with reactive axillary lymph nodes noted at the axillary tail. We were able to proceed with primary closure post left simple mastectomy. The HPE revealed a fairly circumscribed encapsulated breast mass with the proliferation of intralobular stroma, focal areas of stromal overgrowth and exaggerated intracanalicular pattern of the glands. The spindle cells are monomorphic with mild pleomorphism but exhibit several mitoses of about 8/10 hpf (Figure 4). The findings were consistent with a diagnosis of borderline phyllodes tumour. All the margins are clear, and none out of 13 lymph nodes are involved by the tumour.

The patient was subsequently referred to the oncology team and was subjected to 40 Gy in 15 fractions of radiotherapy. The patient was well and had no signs of local recurrences throughout our clinic follow-up. 

## Discussion

It is difficult to differentiate between phyllodes and fibroadenoma based on physical examination, yet radiographically, it is even more challenging to discriminate between them. Histologically, it can be benign, borderline, or malignant. ﻿Phyllodes tumours should be suspected when a patient presents with a large (more than 3 cm), rapidly growing breast swelling that is palpable clinically [1]. The clinical appearance of this patient matches the suspicion of a phyllodes tumour as described. Even though phyllodes tumour can be suggestive of fibroadenoma from imaging, the history of rapid growth and large size should put phyllodes tumour as one of the differentials [1]. ﻿Even though magnetic resonance imaging (MRI) is considered to be extremely sensitive in detecting breast cancer, it is still difficult to differentiate phyllodes tumours from other breast tumour types [3]. Furthermore, ﻿about 20% of phyllodes tumours present as a non-palpable swelling that can only be identified on screening imaging [1]. ﻿In rare cases, phyllodes tumours can also present with blue discolouration, dilated skin veins, skin ulceration, and nipple retraction [3].

Being a rare entity, phyllodes tumours posed a significant challenge in terms of obtaining the diagnosis, especially in differentiating it with cellular fibroadenoma and malignant breast diseases. Therefore, based on the recommendation, triple assessment by clinical, radiological and histological examination should play a pivotal role in arriving at the diagnosis, just like other breast lesions, especially in lesions that are rapidly progressing in size [3,5].

Given the rarity of the disease and based on current studies, simple mastectomy with negative margins is recommended for giant benign phyllodes tumours [3]. ﻿Axillary lymph node involvement due to phyllodes tumours is rare, even when tumours are malignant. Based on the Surveillance, Epidemiology, and End Results Program (SEER) database study, only 8 of 498 women with known lymph node status had involved nodes [1]. ﻿Although palpable axillary lymphadenopathy is mostly reactive and can be identified in up to 20% of patients, involvement of axillary lymph nodes with phyllodes tumour is rare [1]. Therefore, axillary surgery is seldom required in patients with phyllodes tumours [1,3]. Generally, mastectomy is not required for benign phyllodes tumours unless negative margins cannot be achieved or if the tumour is so large that breast-conserving surgery would affect the outcome in terms of cosmetically [1]. 

﻿If an adequate surgical margin cannot be achieved, adjuvant radiotherapy should be administered, even after mastectomy [1,5]. ﻿However, adjuvant radiotherapy for patients with benign phyllodes tumours that are widely excised is not suggested. In contrast, adjuvant radiotherapy is indicated for patients with borderline or malignant phyllodes tumours following surgical excision [1,3]. ﻿Adjuvant chemotherapy is more controversial and ineffective in phyllodes tumours [3]. Based on studies, ﻿patients with benign or borderline phyllodes tumours are usually treated with surgical intervention and chemotherapy is only offered if the tumour is unresectable [1]. Despite the fact that the presence of hormone receptors has been described in 60% to 75% of phyllodes tumours, hormonal treatment is not effective [1,6].

As for this case, we could proceed with primary closure even though there are many techniques to close the large defect. Deep inferior epigastric artery perforator (DIEP) flap is one of the approaches to consider if the defect is too large for primary closure as it provides better tissues coverage without any damage of any muscles [7]. However, as compared to other flap approaches, the DIEP flap technique requires microsurgical skills expertise. 

## Conclusions

Phyllodes tumours are uncommon fibroepithelial breast tumours capable of a diverse range of biological behaviours and should involve multiple disciplinary team management (pathologist, surgeons, oncologist, etc.). The approach and primary management of phyllodes tumour should follow the currently accepted recommendation in terms of the triple assessment, the classification, and surgical intervention options. 
